# 胸腔镜肺叶切除术后肺栓塞诊断及治疗策略

**DOI:** 10.3779/j.issn.1009-3419.2018.10.10

**Published:** 2018-10-20

**Authors:** 昊 徐, 聪颖 郭, 煜 鲁, 临友 张

**Affiliations:** 150000 哈尔滨，哈尔滨医科大学附属第二医院胸外科 Department of Thoracic Surgery, The Second Hospital Affiliated to Harbin Medical University, Harbin 150000, China

**Keywords:** 胸腔镜, 肺叶切除, 肺栓塞, Video-assisted thoracic surgery, Lobectomy pulmonary, Embolism

## Abstract

**背景与目的:**

总结胸腔镜肺叶切除术后肺栓塞患者的临床特征，探讨肺叶切除术后肺栓塞的诊治方法及预防手段。

**方法:**

回顾性分析哈尔滨医科大学附属第二医院胸外科2007年7月-2017年7月间肺叶切除术术后6例肺栓塞患者的临床资料。

**结果:**

6例患者中3例在发病24 h内死亡，3例患者治愈出院。

**结论:**

肺叶切除术后肺栓塞是胸外科少见的术后并发症，诊断困难，病死率高。术前血栓风险评估及术后预防十分重要。

## Abstract (en)

**Background and objective:**

To summarize the clinical features of patients with pulmonary embolism after lobectomy and to explore the methods of diagnosis and treatment of pulmonary embolism after lobectomy.

**Methods:**

The clinical data of 6 patients with pulmonary embolism after lobectomy between July 2007 and July 2017 were retrospectively analyzed.

**Results:**

Of the 6 patients, 3 died within 24 h of onset and 3 patients were cured and discharged.

**Conclusion:**

Pulmonary embolism after lobectomy is a rare postoperative complication in thoracic surgery. It is difficult to diagnose and has a high mortality rate. Preoperative thromboembolic risk assessment and postoperative prevention are important.

肺栓塞（pulmonary embolism, PE）是指来自右心或静脉系统的栓子堵塞肺动脉或其分支而引起的肺循环障碍的临床综合征^[1]^。PE以其起病急骤、高误诊率及漏诊率和高死亡率，越来越引起临床医学界的关注^[2]^。肺栓塞的主要临床表现为突发的胸痛、呼吸困难、咯血等症状，缺乏特异性，更容易被胸外科手术后正常表现掩盖。因此，胸外科术后肺栓塞的诊断更加困难。本研究回顾性分析我科开展胸腔镜肺叶切除手术十年间的肺栓塞病例，总结胸腔镜肺叶切除术后肺栓塞的特点，提高胸外科术后肺栓塞的诊断与治疗水平。

## 1 资料与方法

### 1.1 一般资料

2007年7月-2017年7月哈尔滨医科大学附属第二医院胸外科胸腔镜肺叶切除术后诊断为肺栓塞患者6例，平均年龄64.6岁（56岁-68岁）；术后病理诊断均为肺腺癌，其中右肺上叶4例，右肺下叶1例，左肺下叶1例。诊断主要依靠临床表现，术后出现无法解释的呼吸困难，同时可伴有胸痛、发热或咯血等症状。体格检查可发现呼吸频率增加，心率加快，血压下降以及血氧饱和度降低。实验室检查发现D-二聚体升高以及氧分压降低、二氧化碳分压降低等。辅助检查可发现心电SI、QIII、TIII改变（I导联S波变深，Ⅲ导联出现Q波和T波倒置），电轴右偏及肺性p波等。床旁心脏超声检查发现右心房、右心室增大及肺动脉压力增高等。肺动脉CT血管造影（CT angiography, CTA）检查可发现肺动脉充盈缺损。

### 1.2 治疗

患者诊断考虑为肺栓塞后，嘱患者绝对卧床，吸氧，监测心率血压血氧变化。3例患者行气管插管机械通气，行机械通气患者均行溶栓治疗，方案为：重组组织型纤溶酶原激活物（recombinant tissue plasminogen activator, r-tPA）10 mg/10 min静脉注射，40 mg/2 h或90 mg/2 h维持。3例未行机械通气患者给予抗凝治疗，方案为：低分子肝素抗凝，4, 100 U/12 h，症状改善后逐步改为华法林抗凝，以PT延长至标准的2倍-3倍，即INR 2-3。

## 2 结果

研究发现6例肺栓塞患者均出现不同程度的呼吸困难和胸痛。6例患者中均出现心跳增快，大于100次/分，其中3例患者伴有血压降低，1例患者出现心跳骤停。6例患者实验室检查均出现D-二聚体升高及血气改变。4例患者可见心电图改变。需要机械通气的3例患者中2例为血氧下降，1例为呼吸心跳骤停。此3例患者均存在血流动力学不稳定情况，行床旁心动超声检查发现右心室及右心房增大，床旁心电可见SI、QIII、TIII改变。结合临床表现及辅助检查，考虑为肺栓塞。由于患者状态差，无法行肺动脉CTA检查。与家属交代病情后，决定行溶栓治疗。溶栓后患者均出现引流增多情况，但临床状态改善不明显，在发病24 h内临床死亡。另外3例患者以呼吸困难为主，体格检查可有心率增快，但生命体征尚平稳。行肺动脉CTA检查确诊肺栓塞。由于患者一般状态尚可，仅给予患者抗凝治疗，均顺利出院。

## 3 讨论

肺栓塞是引起院内非预期死亡的主要原因，由于其临床表现缺乏特异性，缺乏简单而准确的确诊方法，使其误诊率、死亡率均较高^[3]^。胸外科肺叶切除术大部分为恶性肿瘤患者、患者年龄较大且手术操作较复杂，术后发生肺栓塞风险大。胸外科医生需要提高肺栓塞的风险意识，对于高危患者早期干预，尽量避免胸外科术后肺栓塞的发生。

肺栓塞的临床症状具有多样性和缺乏特异性的特点，其严重程度与肺动脉堵塞程度、疾病的发展速度以及基础心肺功能相关^[4]^。从无明显症状到严重低氧血症、右心衰竭、心源性休克甚至猝死，均有可能由肺栓塞引起。不明原因的呼吸困难是最常见的症状，本研究6例患者均有不同程度的呼吸困难。胸痛是肺栓塞另一个比较常见的症状，但由于6例患者均行胸腔镜肺叶切除术，术后切口疼痛使得肺栓塞引起的疼痛被掩盖。干湿啰音，胸膜摩擦音，心率加快等症状在术后均容易被忽略。发绀、大汗、血压降低、眩晕等症状出现常预示肺栓塞面积较大，预后较差^[5]^。3例患者出现发绀及血压降低的症状，均使用机械通气治疗。

D-二聚体是肺栓塞诊断的重要排除指标，小于500 μg/L可以排除肺栓塞的可能^[6]^。对于胸科手术患者，术后D-二聚体均有不同程度的升高，此次6例肺叶切除术后患者，D-二聚体均升高明显。动脉血气分析检测对于肺叶切除术后肺栓塞的诊断具有较大的意义。动脉氧分压下降（< 80 mmHg）、肺泡-动脉血氧分压差增加（> 20 mmHg）以及低碳酸血症对于肺栓塞的诊断具有较大的意义^[7]^。心电改变和超声心动在肺栓塞患者中也较为常见，但均不十分特异。肺动脉造影是诊断肺栓塞的金标准，能够显示直径在0.5 mm以上的血管病变^[8]^。但由于肺动脉造影是一种有创操作，存在一定的并发症和风险，临床上常用肺动脉CTA代替肺动脉造影。肺动脉CTA对于肺动脉主干和叶栓塞准确率为97%，段动脉栓塞准确率为68%^[9]^。但肺动脉CTA检查需要一定的准备时间，而且需要将患者转运至扫描间。对于血流动力学不稳定的严重的肺栓塞患者，常常不具备行肺动脉造影或者肺动脉CTA的条件。本组病例，对于生命体征平稳的患者，均行肺动脉CTA检查，获得明确诊断。对于危重的患者，通过症状、查体、实验室检查和辅助检查综合判断，诊断肺栓塞。

肺栓塞的治疗包括抗凝治疗、溶栓治疗、介入治疗和外科手术治疗^[10]^。对于生命体征平稳的患者可以通过吸氧、止痛、强心以及降低肺动脉压的手段治疗。抗凝治疗可以控制血栓进一步加重，并且降低血栓复发风险^[11]^。虽然抗凝治疗不能够溶解血栓，但出血风险也相对较小。对于术后早期患者，溶栓治疗存在严重出血风险时，抗凝治疗是较好的选择。溶栓治疗是通过将患者血液中的纤维蛋白溶解酶原转化为纤维蛋白溶解酶，进而达到溶解血栓的目的。在急性肺栓塞症状出现的48 h内进行溶栓治疗，其治疗效果最好^[12]^。但是对于胸外科术后患者，术后早期溶栓存在一定的出血风险。虽然文献报道胸外科手术不是溶栓治疗的禁忌证^[13]^。但本研究3例肺叶切除后溶栓患者中，均出现较严重的引流增多现象。

胸外科术后肺栓塞是高度致命并发症。肺动脉造影和肺动脉CTA是诊断肺栓塞的金标准，对于无法进行肺动脉CTA检查的患者，结合临床表现和辅助检查，也能做出诊断。对于已经诊断的肺栓塞患者，推荐积极的抗凝或者溶栓治疗。术前仔细评估患者的血栓风险，将患者按风险程度分级并采取不同的预防措施，是预防肺栓塞最好的办法。
